# ‘It is not the State's fault that we have a person like this’: relations, institutions and the meaning of ‘rights’ to carers of People with Psychosocial Disabilities in Chile

**DOI:** 10.1017/gmh.2015.20

**Published:** 2015-11-20

**Authors:** C. R. Montenegro, F. Cornish

**Affiliations:** Department of Methodology, London School of Economics and Political Science, London, UK

**Keywords:** Carers, CRPD, human rights, local meanings, psychosocial disabilities

## Abstract

**Background.:**

The UN Convention on the Rights of Persons with Disabilities (CRPD) has been adopted by national governments to advance the interests and wellbeing of people with psychosocial disabilities (PPSD). It is often assumed that the adoption of a ‘rights’ framework will advance the dignity and autonomy of PPSD. However, little is known about how families and communities understand ‘rights’. The present paper, based on research conducted in Santiago, Chile, takes a contextual approach to rights, asking: How do family carers of PPSD understand and use the idea of ‘rights’? How does the context of caregiving shape families’ understanding of rights?

**Methods.:**

Four focus groups were conducted with a total of 25 family carers (predominantly mothers) of people diagnosed with schizophrenia and other severe neuropsychiatric conditions. Thematic analysis was conducted.

**Results.:**

Carers’ experience of caregiving was marked by isolation, stigmatization, a lack of support and mistreatment by public services. Their family networks did not provide sustained help and support, and the public services they had used were characterized by scarce resources and inadequate support. Carers did not refer to rights of dignity or autonomy. Given an unsupportive context, and worries about who would care for their child after the carer's death, their primary interest in ‘rights’ was a right to guaranteed, long-term care. While carers endorsed the idea of universal, state-supported rights, appeals to compassion and the exchange of favours were spoken of as the most effective strategies for gaining a minimum level of services and support.

**Conclusions.:**

Carers’ understandings, framed against a background of unmet needs and shaped by a history of unsatisfactory interactions with services and institutions, do not resonate with the principles of the CRPD. We suggest an expanded, relational struggle for rights that acknowledges the role of families and the tensions surrounding the distribution of rights within the family.

## Introduction

According to the World Health Organization, globally, neuropsychiatric conditions are responsible for 32% of all disability-adjusted life-years (World Health Organization, 2005). Mental disorders are highly prevalent in Latin America, including high rates of suicide, substance abuse, maternal mental health problems, child and mental health problems and others (Pan American Health Organization, 2010). Yet according to the Pan American Health Organization, public investment in mental health policies and services remains largely insufficient (Pan American Health Organization, 2014), with the percentage of the health budget allocated to mental health still below the European, Eastern Mediterranean and Eastern Pacific Regions of the WHO (World Health Organization, 2011). Transnational efforts to bring dignity and equality to people with psychosocial disabilities (PPSD) have increased over recent years, supported by the proclamation of the Convention on the Rights of Persons with Disabilities (CRPD), which includes disabling psychiatric conditions (United Nations, 2006). This convention has been proclaimed as a powerful global tool for states and civil society to develop inclusive policies and services for persons with disabilities and their families, in the context of the replacement of institutional care with community-based services (Dhanda, 2008; Drew *et al*. 2011; Harpur, 2012; Mittler, 2012).

Since the early 1990s, Chile has made important advances in the process of deinstitutionalization and the development of community-based mental health services (Araya *et al*. 2009). These advances have been accompanied by significant advocacy efforts aimed at the modernization of outdated legal provisions related to mental health and their replacement with new legal frameworks in accordance with the principles of the CRPD (Observatorio de Derechos Humanos de las Personas con Disacapacidad Mental, 2014; World Health Organization & Ministerio de Salud Chile, 2014). Framing the issue of psychiatric and psychosocial disabilities as a matter of ‘rights’ has been central to these efforts, with ‘rights’ becoming part of the normal discourse of policy makers, academics and NGOs.

### The CRPD

The relation between Mental Health and Human Rights is complex and multifaceted, commonly portrayed as bidirectional (Funk & Van Ommeren, 2010). On the one hand, mental health problems increase vulnerability to human rights abuses, such as violence, discrimination and lack of access to basic services. On the other hand, human rights abuses can cause mental health problems. Accordingly, global policy (Funk & Van Ommeren, 2010) has adopted a rights-based approach to understanding and tackling the systematic and underreported abuse and exclusion suffered by people with a psychiatric diagnosis, especially in low- and middle-income countries (Funk & Van Ommeren, 2010; Drew *et al*. 2011).

The CRPD has been promoted as the most comprehensive human rights tool designed to address this ‘unresolved global crisis’ (Drew *et al*. 2011, p. 16). Praised for its effective and inclusive process of design, creation and ratification, it was ‘designed to change society’ (Ito, 2014, p. 101), aiming to ‘promote, protect and ensure the full and equal enjoyment of all human rights and fundamental freedoms by all persons with disabilities, and to promote respect for their inherent dignity’ (United Nations, 2006, p. 5).

Within the field of disabilities, including psychosocial disabilities, the majority of scholarship on ‘rights’ is concerned with the utilization and implementation of the CRPD. Topics addressed include, for example, the local legal and institutional adjustments necessary for implementation of the CRPD (Lawson, 2008; Lord & Stein, 2008; Dinerstein, 2011; Lang *et al*. 2011) and the ways in which different advocacy groups hold governments accountable for its implementation (Harpur, 2012; Mittler, 2012; Wildeman, 2013).

Without denying the need for this type of institutional-level analysis, in this paper we offer a different perspective. On the basis of qualitative research, we explore the local understandings and significance of the idea of ‘rights’ among families and carers of persons living with disabling psychosocial conditions in Chile.

## Conceptual discussion: rights, context and care

Authors from a range of disciplines, and studying different struggles have reflected on the local meanings and dynamics around the idea of human rights (Rabben, 2005; Englund, 2006; Saunders, 2008; de Feyter, 2011; Campbell & Nair, 2014; Unnithan & Pigg, 2014). Together, the contextual approach to rights explicitly moves away from formal and abstract legal analysis, privileging the everyday uses of ideas of justice in action, embracing ‘particularism, paradox and conflicting values’ (Niezen, 2011, p. 683), resonating with broader analysis of the tensions and distances between global prescriptions and local realities in health policy (Campbell *et al*. 2012; Biehl & Petryna, 2013; Burgess, 2014).

As a whole, this line of research highlights the gaps between idealized and abstract formulations of ‘rights’, and the everyday experience of people whose rights are violated. In the field of psychiatric disabilities, Read *et al*. (2009), in documenting abuse of those with severe mental illnesses in Ghana, note a marked contrast between a rights-based approach, heavily reliant on the state as the main driver of policy, and the actual practices through which communities and families deal with their relatives disabilities. To cope, families draw on a form of local morality that places the emphasis on the group and its stability, thus justifying the exclusion of radical difference. ‘Rights talk’ did not resonate with this logic, in a context of minimal state support, and families bearing the enormous demands of long-term care. Campbell & Nair (2014) observe that the concept of ‘women's rights’ had no traction among women in a deeply deprived rural South African community, for whom poverty was considered a more pressing issue than gender relations, and where traditional and NGO leaders expressed no support for rights talk.

As a result, such studies suggest the idea of ‘rights’ simply fails to make an impact at the level of concrete caregiving. Moreover, they claim that people's life circumstances undermine the local significance of the concept of rights (Unnithan & Pigg, 2014).

As well as entailing gaps at the community level, it has been argued that the language of rights fits uneasily at the official or state level. In his analysis of interactions between NGO representatives and the poor in Malawi, Englund (2006) argues that the experts used the discourse of rights to obscure the experiences of claimants and people in need, and that this rhetoric came to be used as a mechanism of distinction between the experts and the poor. In a similar vein, Rebecca Saunders, analysing the work of the South African Truth and Reconciliation Commission states that the ‘deployment of human rights language, refined and standardized into a legalistic technology of rights and the evidentiary information required to be eligible for them, ultimately rendered invisible numerous forms and aspects of suffering and was perhaps particularly deleterious precisely because it claimed to be exposing suffering’ (2008, p. 59).

The field of disability studies has substantially advanced the critique of western conceptualizations of rights, particularly their liberal and Eurocentric underpinnings, their inability to account for the experiences of disabled persons in the global South, and their blindness to the responsibilities of powerful institutions of the global North in the creation of contexts leading to impairment and disability (Meekosha, 2011; Grech & Soldatic, 2015). To clarify the extent to which this criticism resonates with the experience of carers, we explore the meaning of ‘rights’ to family carers of PPSD in Chile. Indeed, in relatively advantaged settings of the global North, a more relational approach to rights has also been gaining attention, given mounting evidence that the wellbeing and interests of disabled people are inextricably bound up with the care, wellbeing, and interests of their families and carers (Pennell *et al*. 2011; Clough, 2014). In this context, the concept of ‘family rights’ is being explored (Melton, 2010; Pennell *et al*. 2011). The current study contributes to this literature an exploration of what ‘rights’ mean to family members. Specifically, this paper asks:

• How do family carers of Persons with Psychosocial Disabilities understand and use the idea of ‘rights’?
• How does the context of caregiving shape families’ understanding of rights?

## Methods

### Context of the study

In Chile, since the early 1990s, Mental Health Care has moved from closed psychiatric hospitals to an array of community-based services, following the vision shared by South American countries in the ‘Caracas Declaration’ (Minoletti *et al*. 2012). This process, oriented towards comprehensive health care, rehabilitation and social inclusion for people with mental health conditions, has made steady progress since then, but funding and staff remain insufficient to address the growing demands of the population, and have allowed only a limited focus on rehabilitation and social inclusion (World Health Organization & Ministerio de Salud Chile, 2014).

Due to these limitations, particularly in the case of rehabilitation and support in the community for the most disadvantaged populations, several NGOs have assumed the roles of advocacy and provision of services. The increased role of the voluntary sector in the provision of health and social services for the poor can be traced back to the neoliberal direction of Chile's social policy, prepared during the dictatorship (1973–1990). The dictatorship introduced a series of radical neoliberal reforms, including the transformation of the health sector, and the split of the health system into a small private sector and an overcrowded and poor public sector. In this model ‘justice meant receiving health care according to individual contribution through direct payment or through freely agreed insurance schemes’ (Missoni & Solimano, 2010, p. 4) This meant a radical departure from the National Health Service developed during the prior decades of democracy (Hadjez-Berrios, 2014).

In 2005, Chile implemented an ambitious plan to provide health care for a number of prioritized diseases. The plan was originally called ‘Universal Access with Explicit Guarantees’ (AUGE according to its Spanish initials), and aimed at tackling the extremely unequal character of health provision by securing access to health intervention as a universal right (Dannreuther & Gideon, 2008). The regime prioritizes problems based on the risk they present and the availability of options, defining the responses, waiting times and rights of patients during the process. Three mental health conditions were on the list: Schizophrenia, Depression and Problematic Substance Abuse. However, the availability of treatment depends on the availability of professionals, and the services are usually limited to narrow pharmacological interventions. As Han (2013, p. 283) states, discussing her ethnographic work on mental health policies in Chile, ‘it is in this parsing out of what can be covered and when, and what cannot, that we begin to see how this system fails to comprehend the lives of the poor’.

It is also important to highlight the split between health services and social welfare benefits, the first assigned to the Ministry of Health and the second by the smaller SENADIS (National Disability Service) for the case of disability. The main benefit for PPSD is a ‘Disability Pension’, for those whose disability is accredited by the Commission of Preventive Medicine and Disability or COMPIN (COMPIN, n.d.). Basic state benefits are only available by having a ‘Disability Credential’. This credential acts as a formal validation of the psychiatric diagnosis. Instead of direct services, the credential allow holders to apply for funds, use dedicated car parking, discounted supportive products, etc. (Robles Farías, 2013).

Under the restricted nature of welfare benefits and the narrow biomedical approach of the health system, the provision of services for the most marginalized sectors, including services offering rehabilitation and social inclusion, have increasingly rested on the actions of the voluntary sector, mostly funded by private donations and to a lesser degree through contracts with the State (Delamaza, 2010). The current study was conducted with carers of persons linked to ‘Fundación Rostros Nuevos’ (New Faces Foundation) and ‘Comunidad Terapeutica de Penalolén’ (A therapeutic community located in a borrow in east Santiago called Penalolén), two of the most active NGOs in the field of psychosocial disabilities (Díaz *et al*. 2011). Both organizations work at a local level, providing services for PPSD, receiving funding from donations and campaigns and from the public health sector, and both frame their action in terms of the protection and promotion of the rights of PPSD (Díaz *et al*. 2011; Fundacion Rostros Nuevos, 2014).

The 25 carers who participated in the focus groups had links with these organizations through the services that their children received at the time of the interviews or before. They were mostly mothers, aged between 38 and 73 years old, whose children were aged between 19 and 55. Most of these children had a diagnosis of schizophrenia or related disorders (15 out of 25). All these families came from disadvantaged contexts within Santiago.

There are no systematic studies of the public understanding of, or stigmatization of PPSD or their carers in Chile. Yang *et al*. (2007) sought to identify aspects of ‘what matters most’ (Yang *et al*. 2007) to Chileans, in the interest of developing culture-specific instruments to evaluate stigma. The long-lasting influence of Catholic values after colonialism introduced strong expectations about gender roles in the family and in society, valuing women's role as devoted mother, responsible for domestic order, and valuing men as economic provider and leader (Yang *et al*. 2013). In a similar vein, studies have discussed the challenges posed by the Chilean cultural context – which is traditionally marked by collectivistic values – for the introduction of individualistic notions of self-determination and autonomy (Marfull-Jensen & Flanagan, 2015). The differentiated roles of mother and father and the limits of autonomy in relation to the group are values that are very pertinent to the experience of caregiving and in the definition of rights, and which will emerge in the discussion of the results, below.

### Research design

A focus group study of family carers’ understandings of rights was undertaken. Based on the constructivist assumption that the social world ‘is actively constructed by people in their everyday lives’ (Gaskell, 2000, p. 39), and given the study's interest in the elaboration and negotiation of meaning from the perspective of actors in context, focus group where employed, especially because the language of rights is relatively new in this context, and unfamiliar to participants. Morgan (1997, p. 11) claims that focus groups ‘may have an advantage for topics that are either habit-ridden or not thought out in detail’. The group setting allowed participants to generate and elaborate meanings together.

### Sampling

The sampling procedure was mainly guided by a theoretical interest in a relevant natural group (Gaskell, 2000): caregivers of adults with psychosocial disabilities living in conditions of poverty and exclusion. Inside this milieu, a convenience sampling approach was used, and the organizations acted as mediators, inviting as much caregivers as possible to be part of the focus groups.

To convene caregivers was a challenging task. In general, caregivers did not have much time for this type of activity, and they were hard to reach. Finally, after significant efforts, 31 caregivers accepted the invitation and 25 showed up, forming four focus groups, two for each organization.

### Data collection

Four focus groups were held, in April 2012, with six to seven participants in each group. Written informed consent was obtained. A topic guide structured the focus groups. The first section started with a general invitation to talk about the experience of caring for a person with a mental disability. The aim was to provide a sense of shared experience, and to foster the emergence of issues that could serve as background to the following topics. The next two topics led the conversation to the everyday challenges and difficulties, and to the consideration of how those difficulties could be reduced.

The second section started by addressing the action of the State. The following topics revolved around the meaning of the idea of rights and the level respect of society regarding those rights, to end with the question of whether or not they themselves respected the rights of their relatives with disabilities.

### Analysis

The conversations generated in the four focus groups where registered in specialized audio recording devices. This recordings where then transcribed in Spanish and the transcriptions were translated to English by the principal investigator, who was able to choose the right words and expression where there was not a direct translation. A mixed approach of inductive and deductive coding was used to analyse the transcriptions (Fereday & Muir-Cochrane, 2008). Using Attride-Stirling's guide (2001), themes were firstly established by the research questions and the general theoretical decisions, and then revised inductively through an iterative process of: (a) coding the data into themes; (b) grouping codes into organizing themes; (c) grouping organizing themes into global themes. This process was aided using Atlas.Ti software.

In the following section, the results of this process of analysis are presented. The first section, ‘The experience of Caregiving’ draws upon the first two global themes, labelled ‘The Experience of Care’ and ‘Support, Interaction and Recognition’. This provides a detailed account of the complexities involved in the experience of caregiving, from the most personal and emotional elements, to more practical and interactive aspects, because this experience shapes the meaning and implications of ideas of justice, including the idea of rights. The second section is based on the third analytical global theme called ‘Making sense of rights’, and directly explores the meanings attached to rights in the context of caregiving.

Finally, it is important to state that coding for ‘rights’ was not simple. The concept of ‘rights’ is typical of legal and policy discourses rather than everyday discourses. While not completely alien, it was not commonly used in the focus groups. The participants’ discussions typically had the form of a testimony, a reconstruction of their lives as carers, using local expressions of suffering rather than the language of human rights (Saunders, 2008). Thus, in the analysis we paid special attention to other words such as ‘fair’, ‘just’, ‘being entitled to’ or ‘deserve’ to indirectly map out the contours and limits of how people conceive of rights.

## Findings

### The experience of caregiving

The experience of caregiving, according to participants, was marked by almost complete absence of support. Positive concepts such as ‘rights’ were barely mentioned, in a context in which rejection and a sense of failure to meet the needs of care-receivers or families predominated.

#### Bereavement and isolation

Carers described their initial response to the psychiatric crisis and diagnosis in terms of ignorance, isolation, loss and guilt. They lacked knowledge about the nature and possible outcomes of the disease, and even its status as such wasn't completely clear. The onset of the disease was considered a shock, in some cases comparable to the loss of a loved one. These feelings were particularly relevant in the context of their relationships with significant others. For example, Angélica (mother) said:

‘They stopped asking me about my son since a long time, his school friends and their mothers. It was just as if he was dead, because they just don't know how to take it. Once, sometime ago, I met a mother whose son had died, and we started to talk, and I realized that, in my case, people acted just as if my son was dead, they don't ask for him anymore’

A sense of raw isolation and absence of understanding thus characterized caregivers’ experience of others’ responses. Moreover, they did not feel qualified to make sense of the diagnosis for others, or even for themselves. Matilde (mother) said:

‘It's very hard because your context, your family, your friends don't understand, and you can't go and try to explain things that you neither quite understand. But you see your son suffering, and you suffer, but you can't quit your job, and then comes the guilt, and the guilt…’

#### Growing demands, growing uncertainty

Families described a sense of continual uncertainty in the context of ongoing and growing demands, especially due to the high economic impact of the disease that in some cases made families lose most of what they had, due to the costs of treatment and the loss of a job due to the demands of care. In this context, uncertainty and exhaustion is permanent. Mariana (mother) says:

‘Everything is too uncertain, everyday (…) one day I satisfy her demands but the next day she has more demands, and the next day is different, you feel like this is a never ending problem, there's no rest’.

Mariana's quotes also highlights the distribution of care inside the family. In most cases the only person accepting responsibility was the mother, even if she was married or had other adult children. This, in some cases, was accepted as a matter of fact, but in others was a source of tensions, distress and guilt.

#### Fragility and death

One of the most striking elements in the testimonies of carers was the deep sense of fragility around their own death. This goes beyond the day-to-day uncertainty that comes from dealing with critical psychotic or other disease-related episodes. This sense of fragility came from the fear of what would happen to their sons/brothers/sisters once they, the only available carers, cease to exist. Jazmín, mother, puts it this way:

‘Now, how many people live on the street, abandoned? And you look at them thinking ‘these lazy lowlifes’. And how many of them are persons with schizophrenia whose parents died, and then the rest of the family didn't take responsibility and just kicked them out onto the streets. I mean, if I die I know that my son is going to be inside a psychiatric institution eternally, or on the street…’

The feeling of fragility and fear of death itself is projected against the background of an un-caring community and un-caring services that are incapable of looking after people with psychiatric disabilities.

#### Family networks

For most of the participants, the traditional sources of informal support are seen as essentially flawed. Friends and even close family, contribute little to the activities of caring, maintaining an attitude that goes from keeping a safe distance to manifest estrangement and rejection. At times this rejection was accepted, rationalized as a normal human reaction. As José, father, states:

‘It's hard for them [PPSD] to be accepted by the rest, it's really hard, because even our families avoid the interaction with them because, as the doctor explained to me, that's normal human behavior, it's not that they're bad persons, it's just to keep their mental wellbeing, because it is too sad for them so they avoid the relation’.

Roberta, talking about her sister, adds:

‘Sometimes I talk with her family, with her husband, sons, but it's as if they still don't accept this responsibility, and she has been living like this for years, it has been hard. My parents are present but… it's always me, they call me for everything, it's depressing’.

This neglect and distancing profoundly marks the emotional aspects of caregiving, substantiating carers’ claims for formal, solid and sustainable support.

#### Institutions and systems of support

The caregivers’ lack of resources, coupled with weak and even damaging relations with family and surrounding communities, place the formal services of the State as the key source of support for carers. Yet all participants had an extraordinarily vivid set of examples of (mostly) failed interactions with these services, fuelling a pessimistic vision of the capacity of the public system to meet their specific demands.

The predominant concern was the failure of the medical system to recognize the specific needs of PPSD, especially in relation to emergency services and general medical procedures. Daniela, mother, exemplifies:

‘We went to all the emergency services and they didn't want to treat him. They sent us elsewhere, but the fact is that there's no place to take them because our sons are special, they are too sensitive to pain, they need special treatment’.

The education system was also described as failing to provide for the specific needs of carers and care-receivers. Participants identified a deep lack of recognition of their needs. As Daniela says, in talking about inequalities in access to after-school childcare for working parents:

‘The other thing is that there are special schools for working mothers, where normal kids can remain until 7 pm. But working mothers of disabled kids don't have access to any such service’.

Finally, participants’ engagement with the criminal justice system entailed some the crudest forms of miscommunication and misrecognition:

‘My child has been arrested by the police three times, he has been beaten and then the cops have asked me “why is he out in the street?” “Well Sir, that's because they do that! They suddenly leave the house.” And I was there without the [disability] credential, having to run back to my house to gather the documents from the psychiatrist to show them to the police’. (Sofía, Mother)

Without formal documents certifying a diagnosis, the PPSD has no particular ‘rights’ for control-oriented systems like the police. In such cases, the State and its powers represented a source of further vulnerability, rather than a source of the recognition of ‘rights’.

### Making sense of rights

While participants spoke of caregiving in terms of isolation and mistreatment, upon prompting, they elaborated ideas of rights. This section presents the different ways they understood rights.

#### Contradictions in the idea of rights: the contested role of the State

‘I think that since we are born, we are born with rights, primarily the right to life, to freedom, and the rights under the constitution of our country. And every person is a human being, whether it is “normal” or if you have a disability, because there are disabled people that are more normal than those who we believe to be normal’. (Amanda, mother)

The universality of the idea of rights and its basic attributes were embraced across the conversations. Rights were spoken of attributes of our humanity, fundamental, and prior to laws or policy. Yet when speaking of the implementation of rights, context and culture emerged as limiting that implementation.

There was a marked contradiction between embracing rights as a universally guaranteed set of standards safeguarded by law and implemented though social policy, and needing to actively claim those rights through an emotional appeal to solidarity. Claudio, brother, expresses these two contrasting positions:

‘We don't need to claim them, because this is stipulated in the constitution. They are Chileans and they need help, it's a moral obligation of the authorities’.

And some moments later:

‘(…) if someone famous, someone that makes a “Telethon” doesn't come to help, this is not going to change. And, as one is not used to going begging for help, one starts to just survive, but not to live. Because if you don't knock at the door, no one is going to open it’.

‘La Teletón’ is a Chilean charity event held yearly since 1978. All Chilean television networks produce a 27-h transmission to raise funds for children with disabilities treated at the ‘Infant Rehabilitation Institute’ of the Fundación Teletón. In the last version, the equivalent of almost 30 million pounds was raised. In this case, Claudio evokes the moral responsibility of the authorities and their obligation to help people in need, such as his brother, but he places his trust in emotional television campaigns and initiatives conducted by a well-respected and charismatic figure, without which he does not expect rights to be respected, or services to be provided.

#### Rights *v*. compassion

Similarly, in their accounts of how they received services, an appeal to compassion seemed much more effective than a claim to rights. Jazmín states:

‘How hard is to get the disability credential? I don't have it, and why? Because the doctor doesn't believe that my son has schizophrenia, ‘you need to talk with the lady’ he said, and the lady tells me ‘You know what? You have to bring all these documents to me’

Amanda (mother): or be crippled, because crippled people receive better treatment

Jazmín: I mean, I have to bring my son with his foot around his neck or something? (…) Why so much bureaucracy, if at the end they are giving us something that we deserve by law’.

In this case, Jazmín refers to the kind of administrative staff in charge of evaluating benefit applications. Again, the experience of rights appeared very contradictory. Participants expressed expectations of their legal rights being met, yet experienced humiliation and obstruction in trying to access those rights, indicating that they are simply not being treated as legal rights by the authorities.

#### Rights within the limits of my care

In some cases, rights were spoken of as rights to care.

‘(…) my son's a baby, so his rights are completely different in relation to every different disabled person, because my son has the right to be cared for by me, to be protected by me, but if I fail, who's going to take care of him?’ (Fabiola, mother)

Here, the only recognizable right of the son is his right to protection and care, and the only person responsible is the mother herself. Against the core principles of global policy tools such as the CRPD, framing the care recipient as a baby is a stark, yet simplified way to make sense of the totality of her role in front of the other, but the idea of rights is still there, disconnected from any form of positive, normative, legal or political content, becoming just a different way to re-name agents and responsibilities *within* the limits of the relation with the recipient of care.

#### Rights as long-term, ‘total’ care

In response to the deep sense of fragility, rights were sometimes considered as a right to a guarantee of care: of carers. Fabiola says:

‘So, if something happens to me, what is going to happen to him? Because the family hides away, the family is not there, nobody is going to take responsibility. We permanently live with that concern. Therefore we really need to press for there to be something formal, something like an institution’.

‘Like an institution’ points to something beyond the organic relations with family and/or communities. A right to support, for carers, given their conditions and experiences, seems to mean institutionally based long-term care for their children, after their own death, as a guarantee of care. Despite policies of de-institutionalization, for participants, the security of institutional care was sorely missed.

## Conclusions

To conclude, we discuss the findings under four subsections, each one ending with the implications for next steps in terms of research, policy and rights activism.

### The contested meaning of rights: families and the CRPD

It has been stated that the CRPD both signals the change from welfare to rights and recognizes autonomy for persons with disabilities, making disability a part of human experience (Dhanda, 2008). Does this vision line up with the experiences of carers? Our findings contravene this alignment of declarations and experiences.

First, carers navigate a practical world in which neither welfare nor rights apparently exists. Information and services are achieved though complex and damaging bureaucracies, and appeals to compassion appear to work better than entitlements as a means of accessing benefits and basic services.

Second, carers fluctuate between a strong claim for external support, and feelings of guilt regarding the intensity of their commitment to care. The emergence of a mental health condition within the family initiates an expansion of caring responsibilities, and at the same time, a focus of those responsibilities on the main carer. The expression of guilt shows the extent to which families have difficulties locating responsibilities beyond themselves, even in their closest family circle. Alone in a context of neoliberal retreat of health services, the concept of rights does not seem to penetrate this dynamic.

Third, for families the idea of rights points to a sense of *personal* stability and security. The right that concerns them is the right to a sense of security about their child's care. This does not necessarily consider the rights *of their child* as a separate, independent human being. Given the complex set of unmet basic needs, their child's right to autonomy is not a concern. In some cases, carers simply equate what their children ‘need’ with the care they can offer their child. And given that what the carer can do is limited by her own fragility, the possibility offered by ‘rights’ is imagined as a possibility of care beyond the carer. This problematizes the foundations of the idea of rights contained in the CRPD: that persons with disabilities have their own aspirations and interests, fundamentally distinct from those of their carers.

These findings have implications for the rights agenda in the field of psychosocial disabilities. For future conceptual work, the findings suggest an urgent need for a reconceptualization of rights, to include the needs of caregivers and families. A full recognition of the complexities of carers’ experiences is the starting point for the construction of a sense of entitlement. For policy, the findings suggest that policy tools such as the CRPD need to be opened up to recognize and give voice to carers’ understandings of and needs for rights and justice. Further research on carers’ understandings of rights and justice will be needed to inform these developments.

### Rights and relations

Within a contextual approach our study brings special attention to rights understood in the context of relations. In almost every process of deinstitutionalization, carers have become the main source of support in the community, saving unquantifiable state resources (Thompson & Doll, 1982). Yet this free workforce risks exhaustion due to inadequate support. Rights-based policies regarding PPSD need to include carers as claimants of rights and special support (Muir & Goldblatt, 2011).

In our focus groups, family carers repeatedly pointed to the relational character of the disability. ‘It's not the problem of the individual, it is a family problem’ said one of the participants, even requesting forms of research that could frame families as a subject of support in their own right. The liberal approach to rights maintains the primacy of the individual (Freeden, 1991) but in the context of care, as Muir and Goldblatt have argued, ‘right holders cannot be understood as individuals separated from interdependent relationships (…) rights must be informed by the relationships that people wish to foster’ (2011, p. 636). Such a reframing of rights is a significant challenge for the ongoing evolution of international human rights declarations related to disability.

Relational conceptions of rights are controversial and challenging. The changing status of families and carers vis-à-vis the rights of persons with disabling mental health conditions has sparked debate, particularly in high-income contexts, over issues of confidentiality, informal coercion towards compliance, rights of autonomy and the impact in mental health policy reform (Goodwin & Happell, 2006; Donnelley & Murray, 2013; English *et al*. 2014). We suggest that further research on how these tensions unfold in different contexts, guided by a relational approach to rights, is a promising area for comparative research in the emergent field of global mental health.

### Locating the tensions between rights

A relational approach to rights needs to be complemented by an examination of the institutional framework of services and policies available for families and PPSD in each local scenario. In a context of abandonment by public health services and welfare mechanisms, the potential of the notion of rights as a useful tool in the hands of caregivers loses much of its meaning. Dignity and autonomy are given little attention or space when apparently more ‘basic’ rights of survival and access to care are threatened. This places the emphasis on families’ right to support in their caregiving role, alongside the rights of PPSD to care, dignity and autonomy. Are we forced to understand these rights as opposed to each other?

The literature on high-income countries has showed to what extent such opposition has been the case, and how those different forms of rights and the subjects they protect cannot be easily reconciled under a single legislative framework or programme for action (Yeates, 2007; Gilbar, 2011). This has also had expression in the complex relation between user-based and family-based activism, and in the different agendas both groups have pursued over time (Morrison, 2005; Crossley, 2006). But to take this kind of opposition as universal or necessary would deny the specificity of the tension and its relation with the institutional and legal context.

Therefore, instead of a clear cut solution to these tensions, we advocate a contextual approach to rights that embraces particularisms, conflicts and paradoxes (Niezen, 2011). This approach should be able to shed light on the detailed unfolding of the tensions between PPSDs and caregivers, between PPSDs and professionals, services, state bureaucracies, broader communities and so on. Understanding the ways in which families deal with services and bureaucracies on the ground at one level helps us uncover the actual and possible dissonances between their experiences and the principles expressed in global legal frameworks such as the CRPD. At another level, it also shows the extent to which the specific articulation of laws and guarantees put in place in each context shapes the tension between the rights of the PPSDs and those of the family. In other words, the specific tensions that arise between the rights of the PPSD and the rights of their carers are dependent on the particular arrangements of formal and informal supports available to PPSD and their families. For advocates for rights-based approaches, the pattern of existing services and supports is a key resource or barrier to the struggle for, and achievement of rights – both those of PPSD and those of families.

### Expanding the struggle for rights

Although our findings, like other research taking a contextual approach to rights, have problematized the application of the CRPD to the situation of PPSDs in Chile, we suggest that these findings may lead to a re-invigoration of an expanded struggle for rights – rather than a dismissal of the notion of rights.

The epistemic potential of a contextualized and relational approach to the meaning and use of the idea of rights can be seen as a form of commitment in favour of the ‘insurrectionary practice’ of rights (Baxi, 2002, p. 10). According to Emmanuel Renault, ‘rather than simply theoretically representing experiences, it is necessary to struggle against cognitive obstructions to victims’ speaking out about injustices and thus to contribute to the elaboration of a framework that enables them to qualify some social experiences as unfair’ (2004, cited in Vielajus & Haeringer, 2011, p. 95). By giving voice to understandings of rights and justice from the perspective of carers in need of rights and justice, this paper has sought to take a step in that direction.

For rights activism, this perspective suggests the value of including the voices of those involved in the concrete support of people experiencing disabling forms of psychosocial distress, including carers, communities and front line professionals from both health and social services. Only the recognition of the value of those voices will pave the way for a meaningful local translation – and not a mere transposition – of the global discourse of rights in the field of psychosocial disabilities.
